# The complete mitochondrial genome of striped large-eye bream, *Gnathodentex aureolineatus* (Teleostei, Lethrinidae)

**DOI:** 10.1080/23802359.2022.2159557

**Published:** 2023-01-08

**Authors:** Minglan Guo, Yongli Gao, Hui Huang

**Affiliations:** a CAS Key Laboratory of Tropical Marine Bio-resources and Ecology; Guangdong Provincial Key Laboratory of Applied Marine Biology, South China Sea Institute of Oceanology, Chinese Academy of Sciences, Guangzhou, China; b Sanya Institute of Ocean Eco-Environmental Engineering, CAS-HKUST Sanya Joint Laboratory of Marine Science Research, Key Laboratory of Tropical Marine Biotechnology of Hainan Province, SCSIO, Sanya, PR China; c Sanya National Marine Ecosystem Research Station; Tropical Marine Biological Research Station in Hainan, Chinese Academy of Sciences, Sanya, China; d Innovation Academy of South China Sea Ecology and Environmental Engineering, Chinese Academy of Sciences, Guangzhou, China; e The center of instruments and determination, South China Sea Institute of Oceanology, Chinese Academy of Sciences, Guangzhou, China

**Keywords:** Striped large-eye bream, mitogenome, phylogenetics

## Abstract

Striped large-eye bream, *Gnathodentex aureolineatus* (Lacepède, 1802), is of high economic value and has important ecological functions in coral reefs. However, the genetic information of this species is quite limited, and there is taxonomical difficulty in the family Lethrinidae. Here, we present the complete mitochondrial genome of *G. aureolineatus* obtained with a long PCR approach and Sanger sequencing. The mitogenome was 16,940 bp in length, consisting of 37 genes (13 protein-coding genes, two ribosomal RNA genes, and 22 transfer RNA genes) and two non-coding regions. Both maximum-likelihood and Bayesian inference phylogenetic trees placed the genus *Gnathodentex* sister to *Monotaxis* within Lethrinidae. These results contribute toward the taxonomy, conservation, and phylogeny of Lethrinidae.

Striped large-eye bream, *Gnathodentex aureolineatus* (Lacepède, 1802), is one of the most common coral reef fishes widely distributed in the Indo-Pacific (Francis and Randall 1993). In China, *G. aureolineatus* has been recorded in South China Sea coral reefs (Shen et al. 1993; Li et al. 2020; Zhang et al. 2021). As one of the major predators in coral reefs (Carpenter and Allen 1989; Skinner et al. 2020), they usually form aggregations and travel on subtidal reef flats, lagoon platforms, or the upper edge of seaward reefs (Lieske and Myers 1994; Carpenter 1997). However, the genetic information is quite limited for *G. aureolineatus*, since only several of their nuclear and mitochondrial genes have been used in phylogenetic analyses (Lautredou et al. 2013; Chen and Borsa 2020; Fabian et al. 2021). In addition, the family Lethrinidae has been considered a taxonomically difficult group (Chen and Borsa 2020; Ramesh et al. 2020; Zhang et al. 2021). To establish genetic data for species identification, conservation, and evolutionary clarification, we aimed to sequence the complete mitogenome of *G. aureolineatus* and constructed the phylogenetic relationships in Lethrinidae.

Three individuals of *G. aureolineatus* were collected in Triton Island (15°47′N 111°12′E), Xisha, China, on the same survey approved by the Animal Care and Ethical Committee of the South China Sea Institute of Oceanology, Chinese Academy of Sciences (Guo et al. 2016). According to WORMS (https://www.marinespecies.org/) and FishBase (https://www.fishbase.de/), we conducted species identification, along with the collection of the dorsal muscles for total genomic DNA extraction. Then, three specimens (Figure 1) were deposited at the South China Sea Tropical Marine Biology Collection, Chinese Academy of Sciences (Minglan Guo, guominglan@scsio.ac.cn) under voucher numbers: SCSTMBC030980-030982.

**Figure 1.**
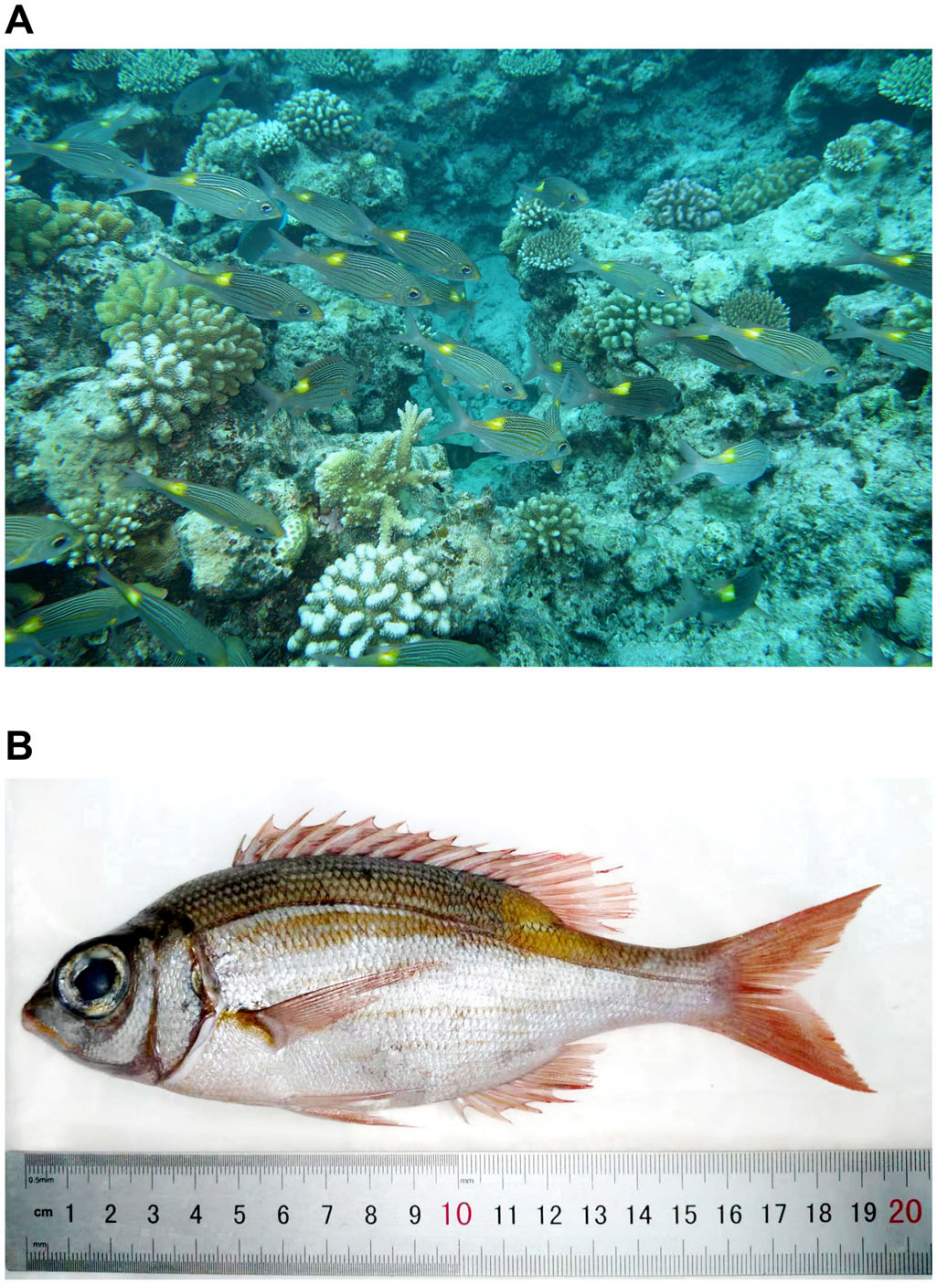
Specimen of *Gnathodentex aureolineatus* (Lacepède, 1802). (A) *G. aureolineatus* found in the coral reefs off Triton Island, Xisha, China (JH Yang and YL Gao). (B) Fixed specimen (ML Guo).

Based on the mitogenome sequences of *Monotaxis grandoculis* (AP009166), *Lethrinus laticaudis* (KU530221), and *L. obsoletus* (AP009165) in Lethrinidae, we designed five pairs of primers (Table 1) to amplify the genes using long PCR for Sanger sequencing (Guo et al. 2016). All sequences were assembled using DNAman software, and the overlaid regions were checked using Sanger sequencing with primers designed from the obtained sequences. The assembled mitogenome (GenBank accession no: OM302214 or NC_063714) was annotated using MITOS (Bernt et al. 2013), aligned with Blastn, and checked with tRNAscan-SE (Chan and Lowe 2019). It was 16,940 bp in length, containing 37 genes (13 protein-coding genes, two rRNA genes, and 22 tRNA genes), the origin of light-strand replication (O_L_), and the control region (D-loop) (Table 2). The GenBank file was used to construct the mitogenome circle map on OrganellarGenomeDRAW (https://chlorobox.mpimp-golm.mpg.de/OGDraw.html) (Figure 2).

**Figure 2.**
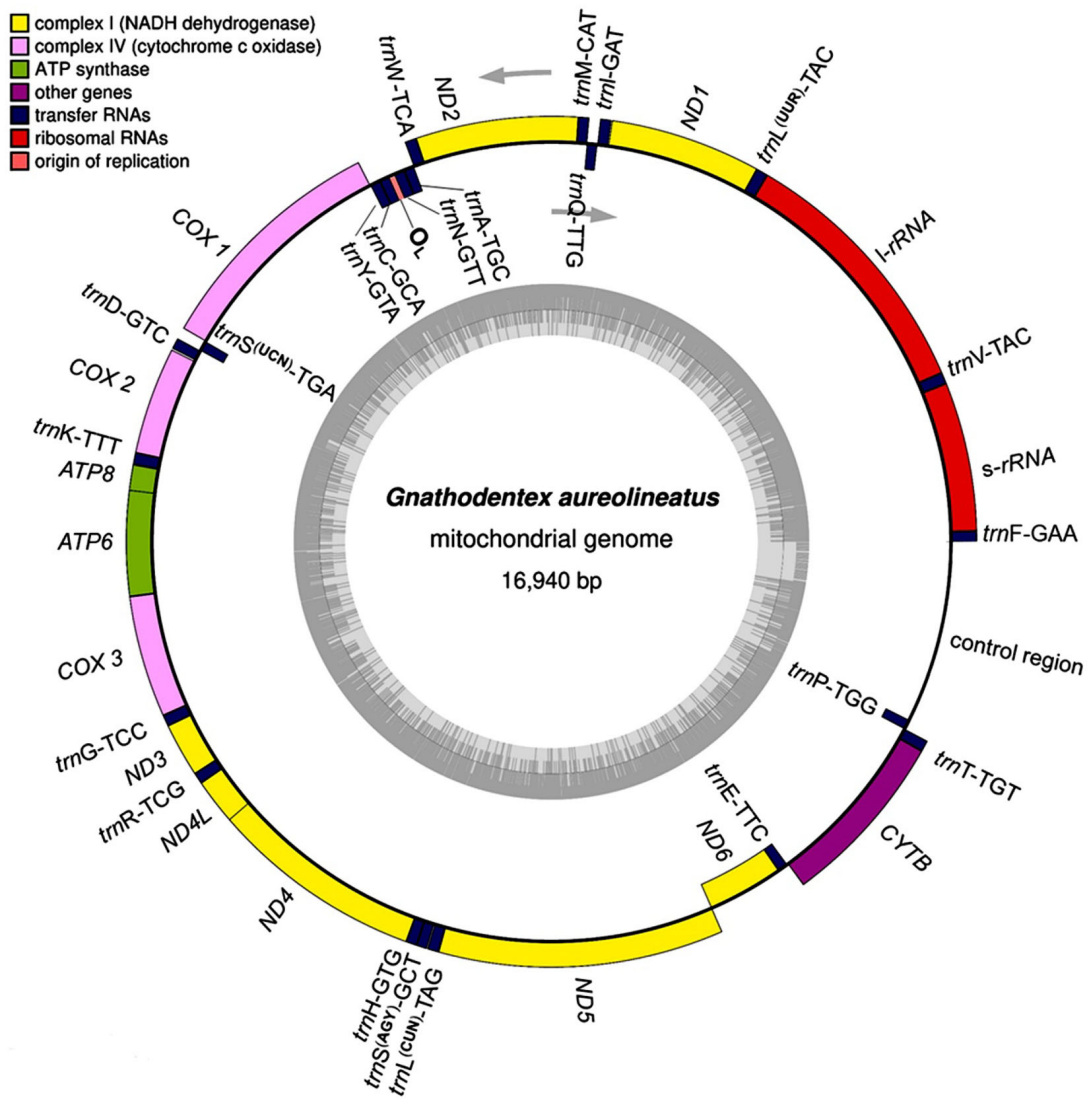
Mitochondrial genome map of *Gnathodentex aureolineatus*. Genes on the heavy and light strands were shown outside and inside the outer circle, respectively. The inner grey ring indicates the GC content. tRNA genes were abbreviated and linked with anti-codons.

**Table 1.** Sequences of primers used in this study.

Primers	Sequence (5’-3’)
GA F1/12S	CACAAAGGTTTGGTCCTGACTTTAC
GA R1/16S	GGGCTCTGCCACCTTAACATGY
GA F2/16S	CTAGTACGAAAGGACCGAGAAGATGA
GA R2/COI	TGTGTGATAAGGRGGMGGGCAGC
GA F3/COI	CGACGRTACTCAGACTACCCHGA
GA R3/ND4L	TTGTAGGAGRTTTARGCTYTGVAGG
GA F4/ND4L	CACCGAACCCACCTWCTCTCYGC
GA R4/Cytb	GCAATTTTTAGTARKGGGTGKGTTTT
GA F5/Cytb	GCCAGGACTYTAACCAGGACTAATG
GA R5/12S	GCGGTGGCTGGCACGAGTTTTAC

The five pairs of primers (GA F1/12S and GA R1/16S, GA F2/16S and GA R2/COI, GA F3/COI and GA R3/ND4L, GA F4/ND4L and GA R4/Cytb, GA F5/Cytb and GA R5/12S) were designed for the mitogenome amplification and Sanger sequencing.

**Table 2.** Characteristics of the mitochondrial genome of *Gnathodentex aureolineatus.*

Locus (abbreviation)	size		Codon		Anti-codon	Intergenic nucleotide^a^	Strand^b^
	Nucleotide (position)	Amino acid	Start	Stop			
*tRNA^Phe^* (*trnF*)	68 (1–68)				GAA	0	H
*12S rRNA* (*s-rRNA*)	958 (69–1027)					0	H
*tRNA^Val^* (*trnV*)	74 (1028–1101)				TAC	0	H
*16S rRNA* (*l-rRNA*)	1698 (1102–2800)					0	H
*tRNA^Leu^*^(^*^UUR^*) (*trnL*)	75 (2801–2875)				TAA	0	H
*ND1* (*ND1*)	975 (2876–3850)	324	ATG	TAA		4	H
*tRNA^Ile^* (*trnl*)	70 (3855–3924)				GAT	0	H
*tRNA^Gln^* (*trnQ*)	71 (3995–3925)				TTG	−1	L
*tRNA^Met^* (*trnM*)	69 (3995–4063)				CAT	0	H
*ND2* (*ND2*)	1046 (4064–5109)	348	ATG	TA–		0	H
*tRNA^Trp^* (*trnW*)	74 (5110–5183)				TCA	0	H
*tRNA^Ala^* (*trnA*)	69 (5252–5184)				TGC	1	L
*tRNA^Asn^* (*trnN*)	73 (5326–5254)				GTT	1	L
O_L_	40 (5328–5367)					−3	–
*tRNA^Cys^* (*trnC*)	68 (5432–5365)				GCA	0	L
*tRNA^Tyr^* (*trnY*)	70 (5502–5433)				GTA	1	L
*COX1* (*COX1*)	1551 (5504–7054)	516	GTG	TAG		1	H
*tRNA^Ser(UCN)^* (*trnS*)	71 (7126–7056)				TGA	1	L
*tRNA^Asp^* (*trnD*)	72 (7128–7199)				GTC	8	H
*COX2* (*COX2*)	694 (7208–7901)	231	ATG	T—		0	H
*tRNA^Lys^* (*trnK*)	73 (7902–7976)				TTT	1	H
*ATP8* (*ATP8*)	168 (7978–8145)	55	ATG	TAA		−10	H
*ATP6* (*ATP6*)	683 (8136–8818)	227	ATG	TA–		0	H
*COX3* (*COX3*)	785 (8819–9603)	261	ATG	TA–		0	H
*tRNA^Gly^* (*trnG*)	72 (9604–9675)				TCC	0	H
*ND3* (*ND3*)	349 (9676–10,024)	116	ATG	T—		0	H
*tRNA^Arg^* (*trnR*)	70 (10,025–10,094)				TCG	0	H
*ND4L* (*ND4L*)	297 (10,095–10,391)	98	ATG	TAA		−7	H
*ND4* (*ND4*)	1381 (10,385–11,765)	460	ATG	T—		0	H
*tRNA^His^* (*trnH*)	69 (11,766–11,834)				GTG	0	H
*tRNA^Ser(AGY)^* (*trnS*)	68 (11,835–11,902)				GCT	4	H
*tRNA^Leu(CUN)^* (*trnL*)	73 (11,907–11,979)				TAG	0	H
*ND5* (*ND5*)	1839 (11,980–13,818)	612	ATG	TAA		−4	H
*ND6* (*ND6*)	522 (14,336–13,815)	173	ATG	TAG		1	L
*tRNA^Glu^* (*trnE*)	69 (14,406–14,338)				TTC	5	L
*CYTB* (*CTYB*)	1141 (14,412–15,552)	380	ATG	T—		1	H
*tRNA^Thr^* (*trnT*)	73 (15,554–15,626)				TGT	−1	H
*tRNA^Pro^* (*trnP*)	69 (15,694–15,626)				TGG	0	L
Control region	877 (15,695–16,940)						–

^a^Numbers correspond to the nucleotides separating different genes. Negative numbers mean overlapping nucleotides between adjacent genes. ^b^H and L indicate genes encoded on the heavy and light strands, respectively.

The gene arrangement in the mitogenome of *G. aureolineatus* was identical to that of the above species in Lethrinidae. *ND6* and eight tRNA genes were transcribed on the light strand, while the other 28 genes were transcribed on the heavy strand. There were two forms of codon recognition in both *tRNA^Leu^* (UUR and CUN) and *tRNA^Ser^* (UCN and AGY) (Figure 2). Moreover, intergenic nucleotides occurred between protein-coding genes and/or tRNA genes. Except for *COX 1*, all protein-coding genes began with the typical start codon ATG. The incomplete stop codon T— appeared in *COX2*, *ND3*, *ND4*, and *CYTB*, and TA– appeared in *ND2*, *ATP6*, and *COX3* (Table 2).

We explored the phylogenies of nine suborder Percoidei mitogenomes available in the NCBI database, including those of *G. aureolineatus* and two outgroup species *Chaetodon auripes* and *Centropyge loricula* (Miya et al. 2001; Yamanoue et al. 2007; Taillebois et al. 2016). All concatenated amino acid sequences of 12 protein-coding genes on the heavy strand were aligned with code constraint under Clustal X (v2.1) (http://www.clustal.org/clustal2/). Then, bootstrapped maximum-likelihood (ML) with 1000 replications and Bayesian Inference (BI) trees were constructed using MEGA 7 (Kumar et al. 2016) and MrBayes v3.2.7 (Huelsenbeck and Ronquist 2001), respectively, based on the result of multiple sequence alignment. The subfamily Monotaxinae (*Gnathodentex* and *Monotaxis*) was separated from Lethrininae (*Lethrinus*) in Lethrinidae (Figure 3). This confirmed the phylogeny in Lethrinidae reported using morphology (Carpenter and Johnson 2002) and nuclear or mitochondrial genes (Chen and Borsa 2020; Lautredou et al. 2013; Fabian et al. 2021).

**Figure 3.**
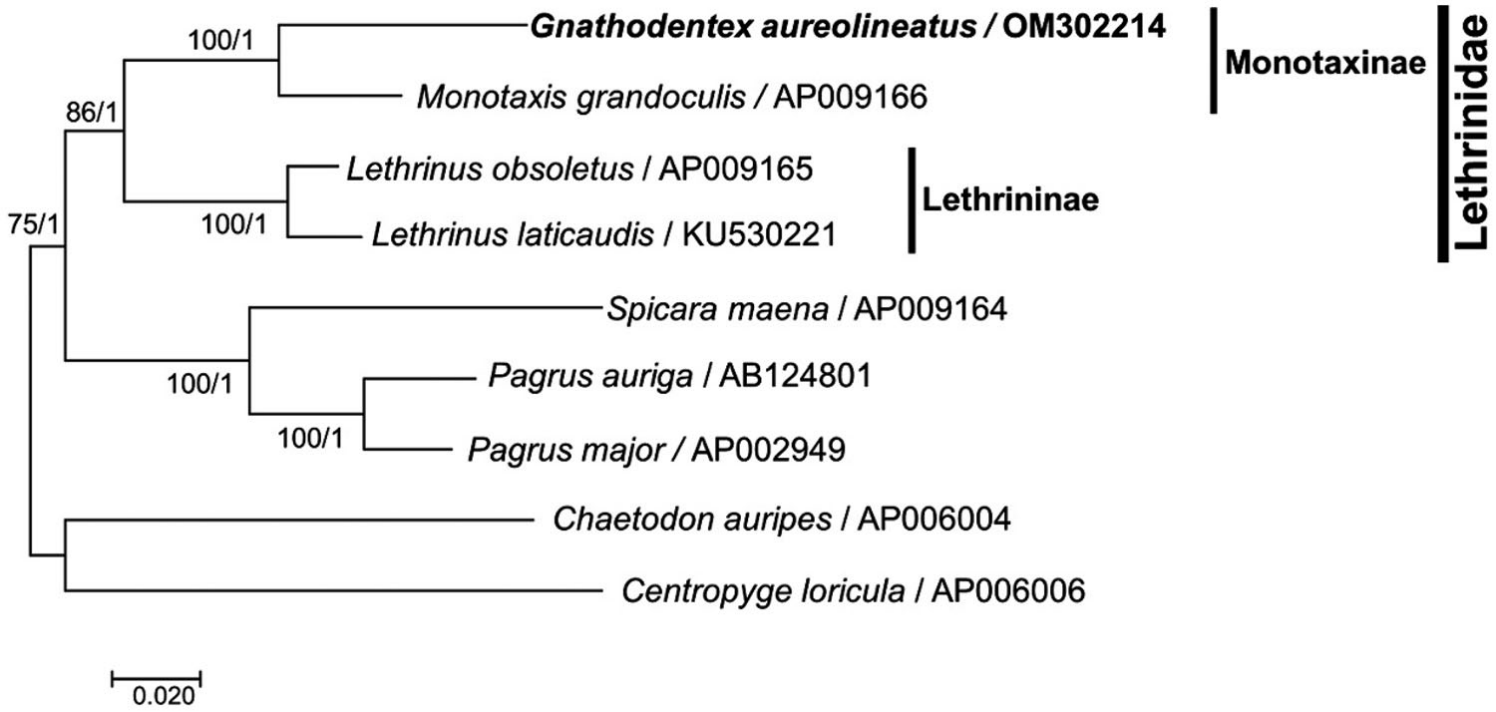
Maximum-likelihood and Bayesian Inference phylogenetic trees constructed with the mitogenomes of nine fish species in the suborder Percoidei, including *Gnathodentex aureolineatus*. The numbers above branches indicate the values of the Maximum-likelihood and Bayesian Inference phylogenetic trees, respectively.

This study provides a new mitogenome and phylogenetic relationship in Lethrinidae. The mitogenome will be useful in delimitating problematic groups in taxonomy. These data will offer genome resources for the conservation of *G. aureolineatus* and a reference for species delimitation and evolutionary research in Lethrinidae.

## Data Availability

The mitogenome sequence data supporting this study are openly available in GenBank with accession no. OM302214 (https://www.ncbi.nlm.nih.gov/nuccore/OM302214.1/) and corresponding RefSeq NC_063714 (https://www.ncbi.nlm.nih.gov/nuccore/NC_063714.1/). The associated BioProject number is PRJNA845703.
